# Development of a Simple Scoring System for Predicting Discharge Safety from the Medical ICU to Low-Acuity Wards: The Role of the Sequential Organ Failure Assessment Score, Albumin, and Red Blood Cell Distribution Width

**DOI:** 10.3390/jpm14060643

**Published:** 2024-06-16

**Authors:** Chang Hwan Seol, Min Dong Sung, Shihwan Chang, Bo Ra Yoon, Yun Ho Roh, Ji Eun Park, Kyung Soo Chung

**Affiliations:** 1 Division of Pulmonology, Allergy and Critical Care Medicine, Department of Internal Medicine, Yongin Severance Hospital, Yonsei University College of Medicine, Yongin 16995, Republic of Korea; seolgoon@yuhs.ac; 2 Division of Pulmonary and Critical Care Medicine, Department of Internal Medicine, Yonsei University College of Medicine, Seoul 03722, Republic of Korea; mdsung@yuhs.ac (M.D.S.); shihwan87@yuhs.ac (S.C.); 3 Department of Internal Medicine, New Korea Hospital, Gimpo 10086, Republic of Korea; borayoon00@naver.com; 4 Biostatistics Collaboration Unit, Department of Biomedical Systems Informatics, Yonsei University College of Medicine, Seoul 03722, Republic of Korea; 5 Department of Pulmonary and Critical Care Medicine, Ajou University School of Medicine, Suwon 16499, Republic of Korea

**Keywords:** intensive care unit, discharge, prediction, SOFA, albumin, red cell distribution width

## Abstract

Despite advancements in artificial intelligence-based decision-making, transitioning patients from intensive care units (ICUs) to low-acuity wards is challenging, especially in resource-limited settings. This study aimed to develop a simple scoring system to predict ICU discharge safety. We retrospectively analyzed patients admitted to a tertiary hospital’s medical ICU (MICU) between July 2016 and December 2021. This period was divided into two phases for model development and validation. We identified risk factors associated with unexpected death within 14 days of MICU discharge and developed a predictive scoring system that incorporated these factors. We verified the system’s performance using validation data. In the development cohort, 522 patients were discharged from the MICU, and 42 (8.04%) died unexpectedly. In multivariate analysis, the Sequential Organ Failure Assessment (SOFA) score (odds ratio [OR] 1.26, 95% confidence interval [CI] 1.13–1.41), red blood cell distribution width (RDW) (OR 1.20, 95% CI 1.07–1.36), and albumin (OR 0.37, 95% CI 0.16–0.84) were predictors of unexpected death. Each variable was assigned a weighted point in the scoring system, and the area under the curve (AUC) was 0.788 (95% CI 0.714–0.855). The scoring system was performed using an AUC of 0.738 (95% CI 0.653–0.822) in the validation cohort of 343 patients with 9.62% of unexpected deaths. When a cut-off of 0.032 was applied, a sensitivity and a specificity of 81.8% and 55.2%, respectively, were achieved. This simple bedside predictive score for ICU discharge uses the SOFA score, albumin level, and RDW to aid in timely decision-making and optimize critical care facility allocation in resource-limited settings.

## 1. Introduction

Despite significant advances in the quality and resources of critical care management, the rate of unexpected mortality after intensive care unit (ICU) discharge remains high, ranging from 5 to 27% [1,2,3,4]. Critical illness burden is increasing because of the rising elderly population, the emergence of pandemics, and the prolonged duration of chronic diseases [5]. Previous studies indicated that critical care professionals adjust their decisions to discharge patients based on the workload and demand for ICU beds [6,7,8,9,10].

Discharging patients from the ICU to lower-acuity wards after aggressive treatment is a critical and complex transition point in patient care. Effective transitions are crucial for maintaining patient safety, optimizing resource utilization, and preserving the quality of care [11]. However, this process is fraught with challenges, particularly the risk of post-transfer deterioration in the patient’s condition, which can result in significant healthcare costs and resource strains. Moreover, such events can adversely affect the relationships between healthcare providers, patients, and their families. A prolonged ICU stay can be a considerable financial burden, leading to significant emotional strain for patients and families, reducing the number of beds available for other patients, and increasing the risk of nosocomial complications [12,13]. Furthermore, the ICU demand has increased compared with the number of available beds, causing an imbalance in the system [14]. This pressure on ICU capacity may significantly affect the decision-making process when discharging patients from ICUs [15]. However, a premature ICU discharge can have significant negative consequences. Patients are vulnerable to readmission to the ICU and may encounter unexpected medical emergencies. These unforeseen ICU readmissions and emergencies are associated with higher hospital mortality rates and extended hospital stays [16,17].

Artificial intelligence-based decision support systems offer potential solutions. However, their implementation is often impractical in settings with limited resources. It is paramount to consider the factors affecting the decision to discharge a patient from the ICU and devise strategies to minimize the mortality rates after ICU care [18]. However, the criteria for making these decisions require more clarity. Many risk scales are not internationally verified, making them difficult to use. This study aimed to propose the development of a short-term mortality prediction score at ICU discharge that is simpler and easier to use at the bedside. This score would be applicable even in resource-limited institutions and has the potential to significantly improve outcomes and resource allocation.

## 2. Materials and Methods

### 2.1. Study Design and Patient Population

We performed a retrospective single-center analysis of patients admitted to the medical ICU (MICU) at a tertiary hospital between July 2016 and December 2021. This period was divided into two phases for model development (July 2016 to April 2018) and validation (June 2019 to December 2021). We developed our model for predicting discharge readiness using the development dataset from July 2016 to April 2018. Subsequently, we aimed to retrospectively validate the developed model’s performance using the validation dataset from June 2019 to December 2021. The Severance Hospital’s institutional review board approved the protocol for development (IRB No. 4-2018-1079) and validation cohorts (IRB No. 4-2021-0820). All studies were conducted in accordance with relevant guidelines and regulations. The need for informed consent was waived due to the retrospective nature of this study.

### 2.2. Data Collection and Clinical Outcomes

In the development cohort, patient data, including age, sex, height, and body weight, were recorded on admission to the MICU. Sequential Organ Failure Assessment (SOFA) scores were evaluated upon admission to the ICU and at discharge to assess illness severity and patient prognosis. Upon ICU admission and discharge, we performed the following laboratory tests: white blood cell (WBC) count, albumin level, C-reactive protein (CRP) level, delta neutrophil index (DNI), and red blood cell distribution width (RDW). In the validation cohort, we assessed the data of patients discharged alive from the MICU, including age, sex, SOFA scores, RDW, and albumin levels at admission and discharge.

The purpose of the discharge scoring system was to predict the survival rates after ICU discharge. This study’s primary outcome was unexpected death within the first 14 days after discharge from the ICU.

### 2.3. Model Development and Validation

We identified the possible predictors of short-term mortality after ICU discharge using univariate logistic regression analysis. Variables with *p* value < 0.05 from the univariate analysis were considered candidates for inclusion in the multivariate logistic regression analysis. A discharge scoring system was developed using multivariate logistic regression analysis. We assessed the predictive power of the multivariate model by checking the C-index and selected a high-performance model.

The calibration plot and Hosmer–Lemeshow goodness-of-fit (H-L) test were used in the calibration. The calibration plot compared the prediction probability obtained from the model with the actual prediction probability. We evaluated the suitability of the discharge scoring system with a reference line close to 45 degrees and a *p* value ≥ 0.05. To validate the discharge scoring system, we calculated the probability, the area under the receiver operating characteristic (AUROC) curve, and the cut-off level of the system using the cohort data of patients discharged from the ICU in different periods. Additionally, the discharge score was compared with the existing prediction score, the stability and workload index for transfer (SWIFT) score.

### 2.4. Statistical Analysis

Patients’ categorical variables are presented as numbers and percentages; continuous variables are presented as means ± standard deviations. The baseline characteristics of the groups were compared using the Mann–Whitney U test for continuous variables and the chi-square test or Fisher’s exact test for categorical variables. We determined the optimal cut-off value for the discharge scoring system using Youden’s index. Statistical analyses were performed using R statistical software, version 3.4.3 (The R Foundation for Statistical Computing, Vienna, Austria).

## 3. Results

### 3.1. Baseline Characteristics in the Development Cohort

**Figure 1:**
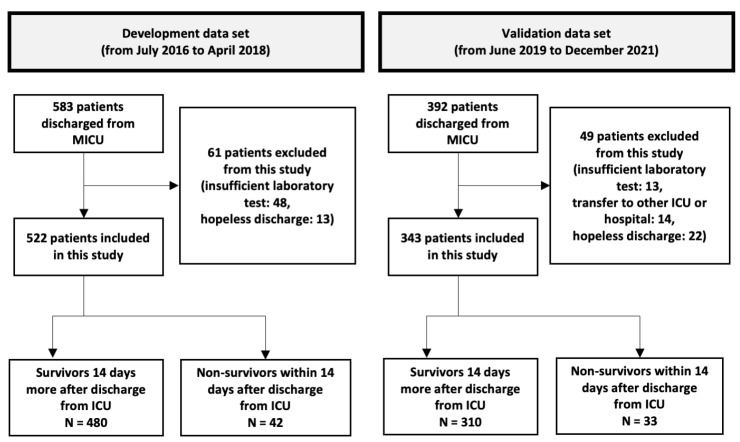
A flow chart showing the inclusion and exclusion of the patients in the study. In the development cohort, we developed discharge scores using cohort data containing 522 patients > 19 years who had been discharged alive from the medical intensive care unit (MICU). We validated the discharge score performance in different periods of the MICU cohort dataset, which was composed of 343 patients > 19 years who were discharged alive from the MICU. MICU, medical intensive care unit; ICU, intensive care unit.

Of the 538 patients discharged from the MICU, 522 were included in the development cohort (Figure 1). Among them, 328 (62.8%) were men, and the mean age was 65.1 years (19–94 years). The number of deaths within 14 days of MICU discharge was 42 (8.04%); these patients were classified as non-survivors. The non-survivors had higher discharge SOFA scores (*p* < 0.001), WBC counts, CRP levels, RDW (*p* < 0.001), and DNI. In contrast, their serum albumin levels were lower (*p* = 0.006). However, the two groups showed no significant differences in WBC count, CRP level, or DNI (Table 1). 

### 3.2. Mortality-Related Factors

The predictors of short-term mortality after ICU discharge were identified using univariate logistic regression analysis (Supplementary Table S1). RDW at admission and at discharge, hospital stay, SOFA score at discharge (SOFA_dis), and albumin level at discharge (albumin_dis) were considered as candidates for inclusion in multivariate logistic regression analysis. The analysis excluded the variable of hospital stay duration because the patients who died had shorter stays.

### 3.3. Development of a Discharge Scoring System

Based on the univariate analysis results, we performed multivariate logistic regression analysis to develop a discharge scoring system. Multivariate model 2, which included SOFA_dis, albumin_dis, and RDW at discharge (RDW_dis), was selected because of its high predictive power with fewer variables than model 1 (C-index: model 1, 0.781 vs. model 2, 0.786) (Supplementary Table S2). The multivariate analysis revealed that SOFA_dis (odds ratio [OR] 1.26, 95% confidence interval [CI] 1.13–1.41, *p* < 0.001), RDW_dis (OR 1.20, 95% CI 1.07–1.36, *p* = 0.003), and albumin_dis (OR 0.37, 95% CI 0.16–0.84, *p* = 0.017) were predictors of unexpected death (Table 2).

**Figure 2:**
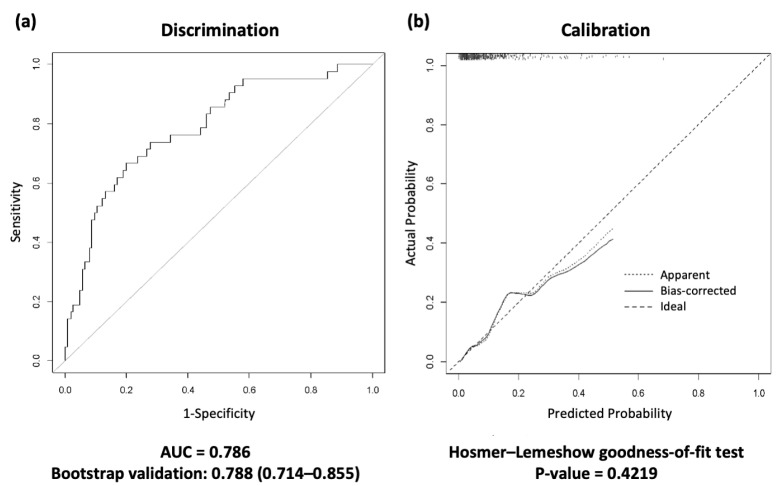
Receiver operating characteristic (ROC) curves for the discharge scoring system in the development cohort. (**a**) The area under the curve (AUC) and 1000 bootstrap samples are presented in the discrimination. (**b**) The calibration plot; the Hosmer–Lemeshow goodness-of-fit (H-L) test was performed in the calibration. A calibration plot graph compares the model’s predicted probabilities with the actual predicted probabilities. This graph should be close to the 45° cutoff and have a *p* value greater than 0.05, indicating that the discharge scoring system predictions are appropriate. ROC, receiver operating characteristic; AUC, area under the curve.

Next, we present the AUROC curve using 1000 bootstrap samples for differentiation (Figure 2). Bootstrap validation was performed using all development datasets because there were few deaths after ICU discharge. Based on these results, a discharge scoring system was developed by assigning weights to the selected variables. The discharge scoring system was defined as follows: 

 Probability = 1/(1 + exp(−A))

A = −4.3634 + 0.1848 × RDW_dis + 0.2345 × SOFA_dis − 1.0040 × albumin_dis

The area under the curve (AUC) and the Hosmer–Lemeshow test showed that the scoring system was well fitted (AUC = 0.788, 95% CI 0.714–0.855, Hosmer–Lemeshow goodness-of-fit test *p* value = 0.422) (Figure 2).

### 3.4. Validation of the Discharge Scoring System

**Figure 3:**
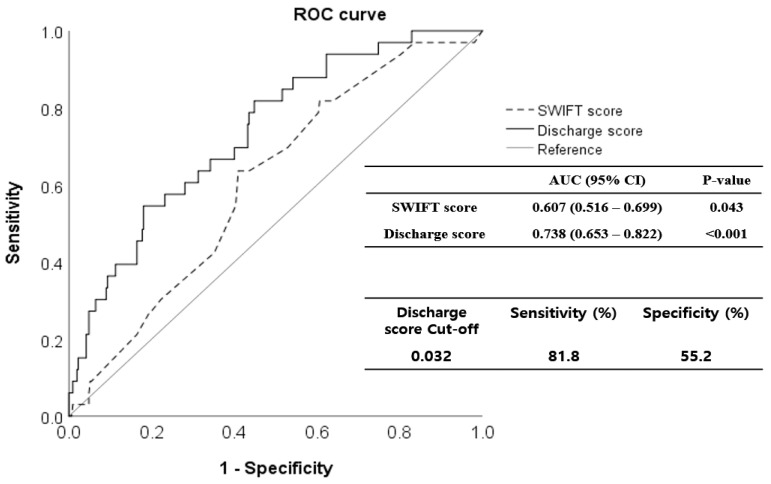
Receiver operating characteristic (ROC) curves for the discharge scoring system in the validation cohort. The discharge score predicted short-term mortality after intensive care unit (ICU) discharge more precisely (area under the curve [AUC], 0.738; 95% confidence interval [CI], 0.653–0.822) than the SWIFT score (AUC, 0.607; 95% CI, 0.516–0.699). When the cut-off value of the discharge score was set to 0.032, sensitivity was 81.8%, and specificity was 55.2%. ROC, receiver operating characteristic; SWIFT, Stability and Workload Index for Transfer; ICU, intensive care unit; AUC, area under the curve; CI, confidence interval.

The validation cohort comprised 343 patients; 231 (67.3%) were men (Figure 1). The mean age of the patients was 68.1 years (range 21–98 years). The mortality rate within 14 days of MICU discharge was 9.62% (33 patients); these patients were classified as non-survivors. In Table 3, the demographic, clinical, and laboratory parameters of survivors and non-survivors are compared. SOFA_dis (*p* < 0.001), RDW_dis (*p* = 0.002), and discharge score (*p* < 0.001) were higher in non-survivors. However, the serum albumin level was lower (*p* = 0.042). In particular, the respiratory (*p* < 0.001), coagulation (*p* = 0.041), hepatic (*p* = 0.003), and cardiac (*p* < 0.001) SOFA scores differed significantly between the non-survivors and survivors. The discharge score showed a higher predictive performance for unexpected death within 14 d of MICU discharge (AUROC 0.738, 95% CI 0.653–0.822, *p* < 0.001) than the SWIFT (AUROC 0.607, 95% CI 0.516–0.699, *p* = 0.043). In the Delong test, AUROCs between the discharge and SWIFT scores differed significantly (*p* = 0.023). When the cutoff value of the discharge score was set to 0.032, the sensitivity and specificity were 81.8% and 55.2%, respectively (Figure 3).

## 4. Discussion

This study aimed to develop a simple bedside ICU discharge prediction scoring system. The findings revealed that SOFA score, albumin level, and RDW were independent predictors of short-term mortality after ICU discharge. The developed discharge score also showed higher performance than the SWIFT score on the day of discharge. This score uses three predictors designed to be readily used even in resource-limited healthcare settings and can significantly improve patient management and resource allocation in critical care environments. By addressing the challenges posed by resource constraints, it can enhance the efficiency and effectiveness of ICU discharge processes, thereby providing a valuable tool for healthcare providers and researchers in critical care.

The ICU, a specialized unit within a medical facility, is equipped with advanced technologies and highly trained staff to deliver intensive and advanced life-support care to critically ill patients. ICU admission and discharge are strictly regulated to conserve limited resources and prevent unnecessary treatments [19]. However, deciding to discharge a patient from the ICU appropriately is a complex and challenging decision because there are no clear and objective guidelines or criteria for determining which patients will benefit from critical care [20].

A few scores are available for predicting unplanned ICU readmission or unexpected death after ICU discharge, such as the SWIFT score, the Sabadell score, the Minimizing ICU Readmission (MIR) score, and the simplified Therapeutic Intervention Scoring System (TISS-28) [4,10,21,22]. The scoring systems’ reported performance varies across studies. Representatively, the SWIFT score, a composite of five items (the original source of ICU admission, ICU length of stay, last partial pressure of oxygen in arterial blood/ fraction of inspiratory oxygen concentration, last Glasgow coma scale, last partial pressure of carbon dioxide), is a recognized scoring system for predicting ICU re-admission and unexpected death. Gagic et al. reported the superiority of the SWIFT score, compared with Acute Physiology and Chronic Health Evaluation (APACHE) III, in predicting unplanned ICU readmission (AUC 0.75 [95% CI, 0.70–0.80] versus AUC 0.62 [95% CI, 0.56– 0.68]) [10]. However, in a prospective cohort study comparing the SWIFT, SOFA, and TISS-28 scores, the three scores showed similar predictive accuracy (the AUC values were 0.66, 0.65, and 0.67, respectively; *p* = 0.58) [23]. Interestingly, the SOFA score was developed for different purposes, and it demonstrated similar accuracy with the SWIFT score in predicting readmission or death after ICU discharge in several studies [23,24]. However, in this study, the discharge score we developed showed superior performance compared to the existing SWIFT score. 

Considering the severity of ICU patients’ conditions, decisions regarding the discharge timing are challenging. Across several studies, the mortality rate after ICU discharge has never been low [25,26], with mortality rates of approximately 10%. Daly et al. reported that premature discharge from the ICU may be associated with increased mortality [27]. In contrast, delayed ICU discharge is linked to extended hospital stays [28] and exposure to multidrug-resistant microorganisms [29], resulting in increased healthcare costs [28]. An excessively delayed ICU discharge complicates the balance between supply and demand within the unit, hindering other patients’ treatments. Thus, physicians must make appropriate adaptations in the triage of waiting patients requiring ICU care when faced with a limited supply of ICU beds.

The decision to transfer patients from the ICU to low-acuity wards involves collaboration among medical staff, patients, and their caregivers. A critical concern is the potential deterioration of these relationships due to adverse events such as post-transfer mortality. Recent advancements have led to the development of artificial intelligence-based patient prediction systems. However, their application remains challenging in resource-limited healthcare settings [30]. Therefore, simple bedside prediction scores that the medical staff can easily use can significantly assist in informed ICU admission and discharge decisions. 

In the development cohort, we identified independent predictors of short-term mortality after ICU discharge, including RDW, albumin level, and SOFA scores. First, the RDW parameter indicates variations in the size of red blood cells [31]. Previous studies identified RDW as a reliable indicator of inflammation [32]. Pro-inflammatory cytokines like TNF-α, IL-6, and IL-1β, present in patients with systemic inflammatory response syndrome (SIRS), impeding erythrocyte maturation, leading to the influx of larger, new reticulocytes into the bloodstream, and increasing the RDW [33,34]. In addition to this, proinflammatory cytokines can directly hinder red blood cell circulation and the flexibility of the red blood cell membrane, which can increase the RDW [35,36]. These findings provide a scientific basis for using RDW as an inflammatory marker in critical illnesses. RDW has been used as an additional marker of many diverse diseases, such as cardiovascular disease, kidney injury, bowel disease, sepsis, and septic shock [37,38,39,40,41,42,43,44]. Fujita et al. reported that RDW is a powerful predictor of mortality in patients hospitalized in the ICU and after ICU discharge [45,46]. Otero et al. also noted that RDW was associated with ventilator-free days and ICU mortality [47,48]. Therefore, RDW may be a valuable indicator for predicting the prognosis of ICU patients upon discharge [49,50].

Furthermore, albumin plays a crucial role in various physiological functions, e.g., by contributing to osmotic pressure, transporting and binding many molecules, acting as an effective plasma buffer, having an antioxidant function, and maintaining microvascular integrity [51,52]. However, several aspects of albumin physiology are altered in critically ill patients. The serum albumin levels are often low in critically ill patients owing to multiple factors, such as malnutrition, increased loss of albumin, impaired hepatic albumin synthesis, and increased capillary leakage [53,54]. Previous studies reported that hypoalbuminemia is associated with adverse outcomes in critically ill patients [55,56,57,58,59]. A meta-analysis of cohort studies and controlled trials evaluated the effects of hypoalbuminemia on the outcomes of acutely ill patients. This study found that hypoalbuminemia was a significant dose-dependent predictor of unfavorable outcomes. Specifically, a 10 g/L reduction in serum albumin levels was found to increase the likelihood of mortality, morbidity, and prolonged stay in the ICU by 137%, 89%, and 28%, respectively [56]. The serum albumin levels are useful predictors of mortality and other outcomes in critically ill patients.

The European Society of Intensive Care Medicine introduced the SOFA score in 1994 to assess the severity of organ dysfunction in critically ill patients. Given that multiorgan dysfunction has been identified as a significant cause of death in ICU patients, the SOFA score has long been used as a predictor of mortality [60,61]. An increase in the SOFA score by two or more points in adults admitted to the ICU with suspected infection demonstrated greater prognostic accuracy for in-hospital mortality than the SIRS criteria or quick SOFA [62]. The sequential assessment of organ dysfunction in the ICU is a good indicator of prognosis [63,64]; in this sense, the SOFA score is a valuable indicator.

Based on these findings, we developed an easy-to-apply ICU discharge scoring system by analyzing ICU patient data to construct a system based on RDW, albumin, and SOFA scores. The developed discharge scoring system provides rapid information and identifies the risks associated with medical ICU discharge. Furthermore, the discharge score system’s performance was maintained in the validation cohort across different periods. The discharge scoring system developed in our research will create an environment where critically ill patients leaving the ICU can receive safer treatment.

Despite its strength in simplifying bedside ICU discharge decisions, this study has some limitations. First, this was a single-center retrospective cohort study, which makes the findings difficult to generalize. In addition, this study was conducted only in a MICU. The results may not apply to patients in different types of ICUs or hospital settings with other patient characteristics. Therefore, rigorous external and prospective validation studies are required in the future. Second, because this was a retrospective study, the ICU discharge criteria applied by the involved clinicians varied. This factor can lead to significant differences in patient condition upon discharge from the ICU. Third, ICU readmission is a crucial factor among ICU quality control indicators. Our study had very few re-admission cases in the ICU; therefore, we could not examine these cases. Fourth, our study solely focused on predicting the survival rates after ICU discharge. The scoring system we used does not consider ethical issues.

## 5. Conclusions

This study proposes a simple bedside-applicable scoring system to predict the safety of ICU discharge using RDW, albumin, and SOFA scores. The scoring system includes factors easily measured in laboratory studies and clinical parameters. By measuring albumin, RDW, and SOFA scores, which are commonly assessed upon ICU discharge, mortality can be predicted 14 days after ICU discharge, enabling efficient medical resource management and patient care. We hope that our research findings will help manage critically ill patients.

## Figures and Tables

**Table 1:** Clinical characteristics of the development cohort.

Variables	Survivors after ICU Discharge (*n* = 480)	Non-Survivors after ICU Discharge (*n* = 42)	*p*-Value
Age (year)	65.0 ± 14.5	66.8 ± 10.9	0.431
Sex (male)	299 (62.2%)	29 (69.0%)	0.294
BMI	22.5 ± 5.5	22.7 ± 3.6	0.773
Charlson comorbidity index at ICU admission	2.98 ± 2.3	3.7 ± 3.0	0.055
Comorbidity at ICU admission			
Congestive heart failure	35 (7.3%)	6(14.3%)	0.127
Coronary arterial disease	68 (14.2%)	9 (21.4%)	0.253
Chronic pulmonary disease	53 (11.0%)	3 (7.1%)	0.605
Chronic kidney disease	115 (24.0%)	10 (23.8%)	1.000
Chronic liver disease	64 (13.3%)	13 (31.0%)	<0.005
Cerebrovascular disease	77 (16.0%)	4 (9.5%)	0.373
Solid cancer	122 (23.4%)	17 (40.5%)	0.044
Hematologic malignancy	22 (4.6%)	3 (7.1%)	0.442
ICU length of stay (days)	10.2 ± 11.7	13.3 ± 12.5	0.100
at ICU admission			
ARDS, *n* (%)	33 (6.9%)	3 (7.1%)	1.000
AKI, *n* (%)	108 (22.5%)	9 (21.4%)	1.000
Septic shock, *n* (%)	125 (23.9%)	8 (19.0%)	0.362
Treatment during ICU stay			
Ventilator, *n* (%)	268 (55.8%)	24 (57.1%)	1.000
Tracheostomy, *n* (%)	87 (18.1%)	4 (9.5%)	0.204
ECMO, *n* (%)	11 (2.3%)	2 (4.8%)	0.281
CRRT, *n* (%)	116 (24.2%)	21 (50.0%)	0.001
SOFA score			
At admission	7.5 ± 3.5	8.7 ± 3.2	0.028
At discharge	5.1 ± 3.1	8.0 ± 3.3	<0.001
Laboratory (at admission)			
WBC (10^3^/µL)	14.3 ± 11.1	13.8 ± 10.1	0.774
CRP	126.6 ± 113.3	105.5 ± 113.5	0.279
Albumin (g/dL)	2.9 ± 6.5	2.6 ± 0.5	0.698
DNI (%)	7.4 ± 12.1	5.0 ± 7.8	0.203
RDW (%)	15.2 ± 2.5	16.4 ± 2.7	0.001
Laboratory (at discharge)			
WBC (10^3^/µL)	9.8 ± 7.4	11.3 ± 7.3	0.203
CRP	66.6 ± 70.4	79.7 ± 68.1	0.247
Albumin (g/dL)	2.7 ± 0.4	2.5 ± 0.4	0.006
DNI (%)	2.5 ± 4.0	3.5 ± 4.4	0.111
RDW (%)	15.6 ± 2.3	17.4 ± 2.8	<0.001

BMI, body mass index; ICU, intensive care unit; ARDS, acute respiratory distress syndrome; AKI, acute kidney injury; ECMO, extracorporeal membrane oxygenation; CRRT, continuous renal replacement therapy; SOFA, Sequential Organ Failure Assessment; WBC, white blood cell; CRP, C-reactive protein; DNI, delta neutrophil index; RDW, red cell distribution width.

**Table 2:** Multivariate logistic regression.

Variables	OR (95% CI)	*p*-Value
SOFA at discharge	1.26 (1.13–1.41)	<0.001
RDW at discharge	1.20 (1.07–1.36)	0.003
Albumin at discharge	0.37 (0.16–0.84)	0.017

SOFA, Sequential Organ Failure Assessment; RDW, red cell distribution width; OR, odds ratio; CI, confidence interval.

**Table 3:** Clinical characteristics of the validation cohort.

Variables.	Survivors (*n* = 310)	Non-Survivors (*n* = 33)	*p*-Value
Age (year)	67.8 ± 13.6	71.1 ± 12.1	0.177
Sex (male)	206 (66.5%)	25 (75.8%)	0.332
ICU admission from ER	214 (69.0%)	21 (63.6%)	0.556
SOFA at admission	8.1 ± 3.7	9.6 ± 4.1	0.032
Discharge Parameter			
Total SOFA	4.1 ± 2.6	6.9 ± 3.8	<0.001
Respiratory SOFA	1.3 ± 0.9	2.2 ± 0.9	<0.001
Coagulation SOFA	0.7 ± 1.0	1.1 ± 1.1	0.041
Hepatic SOFA	0.4 ± 0.9	0.9 ± 1.4	0.003
Cardiac SOFA	0.3 ± 1.0	1.3 ± 1.7	<0.001
CNS SOFA	0.6 ± 0.9	0.7 ± 0.9	0.163
Renal SOFA	0.7 ± 1.1	0.7 ± 1.1	0.341
Glasgow coma scale	14.0 ± 1.9	13.7 ± 1.8	0.566
ICU length of stay	26.2 ± 73.0	38.0 ± 117.6	0.407
Laboratory at discharge			
Albumin (g/dL)	3.0 ± 0.4	2.8 ± 0.4	0.042
RDW (%)	16.0 ± 2.6	17.5 ± 3.1	0.002
P/F ratio	324.4 ± 106.2	235.6 ± 87.3	<0.001
PaCO_2_	36.2 ± 7.6	38.6 ± 11.6	0.244
SWIFT score	20.8 ± 9.9	24.4 ± 8.9	0.043
Discharge score	0.052 ± 0.063	0.139 ± 0.176	<0.001

SOFA, Sequential Organ Failure Assessment; CNS, central nervous system; RDW, red cell distribution width.

## Data Availability

The datasets used and/or analyzed in the current study are available from the corresponding author upon reasonable request. The sources and data used in this study can be deposited publicly.

## Supplementary Materials

The following supporting information can be downloaded from: https://www.mdpi.com/article/10.3390/jpm14060643/s1, Table S1: Demographic data of the development cohort; Table S2: Univariate and multivariate logistic regression for death until 14 days after discharge.
