# Supplementary material for: Development of a Simple Scoring System for Predicting Discharge Safety from the Medical ICU to Low-Acuity Wards: The Role of the Sequential Organ Failure Assessment Score, Albumin, and Red Blood Cell Distribution Width
Source: J Pers Med. 2024 Jun 16;14(6):643. doi: 10.3390/jpm14060643 (PMC11204447; doi:10.3390/jpm14060643)
Supplement: Supplementary file 1 [file jpm-14-00643-s001.zip › jpm-2962536-supplementary.pdf]

Supplementary Table S1. Demographic in development cohort

variable	Death until 14 days after ICU discharge		
	survival (n=480)	death (n=42)	p-value
Age	65.00 ± 14.48	66.81 ± 10.89	0.431
Sex (female)	181 (39.18)	13 (30.95)	0.294
Body weight	59.69 ± 15.71	60.24 ± 9.42	0.823
Height	163.02 ± 9.66	162.95 ± 6.25	0.967
BMI	22.45 ± 5.54	22.70 ± 3.55	0.773
PLT at admission	172.54 ± 121.99	144.26 ± 128.25	0.153
Cr at admission	1.99 ± 2.36	2.00 ± 1.75	0.964
SOFA at admission	7.49 ± 3.48	8.73 ± 3.15	0.028
ALB at admission	2.94 ± 6.45	2.55 ± 0.50	0.698
BUN at admission	36.69 ± 27.39	42.61 ± 22.01	0.174
WBC at admission	14.29 ± 11.12	13.78 ± 10.11	0.774
LAC at admission	2.98 ± 3.10	3.83 ± 3.71	0.128
PCT at admission	16.71 ± 34.85	9.73 ± 18.76	0.288
CRP at admission	126.59 ± 113.26	105.54 ± 113.46	0.279
DNI at admission	7.37 ± 12.05	4.96 ± 7.79	0.203
RDW at admission	15.15 ± 2.46	16.43 ± 2.70	0.001
PaO_2 at admission	101.86 ± 39.70	100.15 ± 42.51	0.790
$PaCO_2$ at admission	36.33 ± 19.12	36.72 ± 15.01	0.897
pH at admission	7.40 ± 0.08	7.43 ± 0.32	0.121
HCO_3 at admission	21.98 ± 6.27	21.47 ± 5.87	0.613
SOFA at discharge	5.12 ± 3.05	7.95 ± 3.34	<0.001
CRP at discharge	66.60 ± 70.44	79.73 ± 68.11	0.247
ALB at discharge	2.68 ± 0.43	2.49 ± 0.39	0.006
DNI at discharge	2.47 ± 3.99	3.50 ± 4.40	0.111
RDW at discharge	15.63 ± 2.25	17.35 ± 2.81	<0.001
WBC at discharge	9.78 ± 7.39	11.29 ± 7.33	0.203
ICU day	9.99 ± 11.37	13.07 ± 12.36	0.095
Hospital day	44.16 ± 45.91	18.64 ± 13.43	<0.001

BMI, body mass index; PLT, platelet; Cr, creatinine; SOFA, Sequential Organ Failure Assessment; ALB, albumin; BUN, blood urea nitrogen; WBC, white blood cell; LAC, lactate; PCT, procalcitonin; CRP, C-reactive protein; DNI, delta neutrophil index; RDW, red cell distribution width; PaO_2, partial pressure of oxygen; $PaCO_2$, partial pressure of carbon dioxide; pH, potential of hydrogen; HCO_3, bicarbonate.

Supplementary Table S2. Univariable and multivariable logistic regression for the death until 14 days after discharge

Variable	Univariable		Multivariable 1 (Model 1)		Multivariable 2 (Model 2)	
	OR(95%CI)	p-value	OR(95%CI)	p-value	OR(95%CI)	p-value
Age	1.009(0.986 ,1.033)	0.4301	1.029(0.938 ,1.128)	0.5483		
Sex	0.696(0.352 ,1.374)	0.2964				
Body weight	1.002(0.983 ,1.022)	0.8224	0.947(0.836 ,1.071)	0.3856		
Height	0.999(0.966 ,1.033)	0.967				
BMI	1.008(0.955 ,1.063)	0.7721				
PLT at admission	0.998(0.995 ,1.001)	0.1521				
Cr at admission	1.003(0.877 ,1.148)	0.9635				
ALB at admission	0.698(0.367 ,1.327)	0.2724				
BUN at admission	1.007(0.997 ,1.017)	0.177				
WBC at admission	0.995(0.965 ,1.027)	0.7731				
LAC at admission	1.069(0.979 ,1.166)	0.1353				
PCT at admission	0.991(0.975 ,1.008)	0.2914				
CRP at admission	0.998(0.995 ,1.001)	0.2803				
DNI at admission	0.977(0.942 ,1.013)	0.2111				
RDW at admission	1.228(1.084 ,1.391)	0.0012	1.027(0.852 ,1.237)	0.7824		
PaO_2 at admission	0.999(0.991 ,1.007)	0.7894				
$PaCO_2$ at admission	1.001(0.985 ,1.017)	0.8965				
pH at admission	3.506(0.577 ,21.309)	0.173				
HCO_3 at admission	0.986(0.935 ,1.04)	0.6119				
SOFA at admission	1.11(1.01 ,1.22)	0.0295	0.947(0.836 ,1.071)	0.3856		
SOFA at discharge	1.32(1.189 ,1.466)	<0.0001	1.281(1.128 ,1.454)	0.0001	1.264(1.132 ,1.412)	<0.0001
CRP at discharge	1.002(0.998 ,1.006)	0.2487				

ALB at discharge	0.324(0.146 ,0.72)	0.0057	0.4(0.175 ,0.913)	0.0296	0.366(0.16 ,0.837)	0.0172
DNI at discharge	1.048(0.988 ,1.111)	0.1222				
RDW at discharge	1.289(1.149 ,1.446)	<0.0001	1.2(1.004 ,1.435)	0.0449	1.203(1.065 ,1.358)	0.0029
WBC at discharge	1.019(0.988 ,1.05)	0.2307				

BMI, body mass index; PLT, platelet; Cr, creatinine; SOFA, Sequential Organ Failure Assessment; ALB, albumin; BUN, blood urea nitrogen; WBC, white blood cell; LAC, lactate; PCT, procalcitonin; CRP, C-reactive protein; DNI, delta neutrophil index; RDW, red cell distribution width; PaO_2, partial pressure of oxygen; $PaCO_2$, partial pressure of carbon dioxide; pH, potential of hydrogen; HCO_3, bicarbonate.
